# Hypomethylation of *CLDN4* Gene Promoter Is Associated with Malignant Phenotype in Urinary Bladder Cancer

**DOI:** 10.3390/ijms23126516

**Published:** 2022-06-10

**Authors:** Fumisato Maesaka, Masaomi Kuwada, Shohei Horii, Shingo Kishi, Rina Fujiwara-Tani, Shiori Mori, Kiyomu Fujii, Takuya Mori, Hitoshi Ohmori, Takuya Owari, Makito Miyake, Yasushi Nakai, Nobumichi Tanaka, Ujjal Kumar Bhawal, Yi Luo, Masuo Kondoh, Kiyohide Fujimoto, Hiroki Kuniyasu

**Affiliations:** 1 Department of Molecular Pathology, Nara Medical University, 840 Shijo-cho, Kashihara 634-8521, Nara, Japan; mae_fumi0107@yahoo.co.jp (F.M.); masaomikuwada@gmail.com (M.K.); ngsoccer11n1212lv@gmail.com (S.H.); nmu6429@yahoo.co.jp (S.K.); rina_fuji@naramed-u.ac.jp (R.F.-T.); m.0310.s.h5@gmail.com (S.M.); toto1999-dreamtheater2006-sms@nifty.com (K.F.); pt_mori_t@yahoo.co.jp (T.M.); brahmus73@hotmail.com (H.O.); bhawal2002@yahoo.co.in (U.K.B.); 2 Department of Urology, Nara Medical University, 840 Shijo-cho, Kashihara 634-8522, Nara, Japan; tintherye@gmail.com (T.O.); makitomiyake@yahoo.co.jp (M.M.); nakaiyasusiuro@live.jp (Y.N.); sendo@naramed-u.ac.jp (N.T.); kiyokun@naramed-u.ac.jp (K.F.); 3 Department of Pharmacology, Saveetha Dental College, Saveetha Institute of Medical and Technical Sciences, Chennai 600077, India; 4 Jiangsu Province Key Laboratory of Neuroregeneration, Nantong University, 19 Qixiu Road, Nantong 226001, China; lynantong@hotmail.com; 5 Drug Innovation Center, Graduate School of Pharmaceutical Sciences, Osaka University, 6-1 Yamadaoka, Suita 565-0871, Osaka, Japan; claudindds@gmail.com

**Keywords:** claudin-4, promoter methylation, bladder cancer, stemness, non-tight junction claudin

## Abstract

The tight junction (TJ) protein claudin-4 (CLDN4) is overexpressed in bladder urothelial carcinoma (BUC) and correlates with cancer progression. However, the mechanism of CLDN4 upregulation and promotion of malignant phenotype is not clear. Here, we analyzed 157 cases of BUC and investigated the hypomethylation of CpG island in the *CLDN4* promoter DNA and its correlation with cancer progression. In hypomethylated cases, CLDN4 expression, cell proliferation, stemness, and epithelial-mesenchymal transition were increased. Treatment of three human BUC cell lines with the demethylating agent aza-2′-deoxycytidine (AZA) led to excessive CLDN4 expression, and, specifically, to an increase in CLDN4 monomer that is not integrated into the TJ. The TJ-unintegrated CLDN4 was found to bind integrin β1 and increase stemness, drug resistance, and metastatic ability of the cells as well as show an anti-apoptosis effect likely via FAK phosphorylation, which reduces upon knockdown of CLDN4. Thus, CLDN4 is overexpressed in BUC by an epigenetic mechanism and the high expression enhances the malignant phenotype of BUC via increased levels of TJ-unintegrated CLDN4. *CLDN4* promoter DNA methylation is expected to be a novel indicator of BUC malignant phenotype and a new therapeutic target.

## 1. Introduction

Claudin-4 (CLDN4) is a major structural protein of epithelial tight junctions and is involved in epithelial differentiation, polarity maintenance, and substantial transport [1,2]. However, in epithelial malignancies overexpressing CLDN4, such as bladder urothelial carcinoma (BUC), colon cancer, gastric cancer, and pancreatic cancer, the barrier function of CLDN4 maintains the tumor microenvironment, retains growth factors, and protects tumors from intratumoral permeation of anti-cancer drugs [3,4,5,6]. CLDN4 expression is correlated to cancer progression in these cancers [3,4,5,6]. The association of CLDN4 overexpression with carcinogenesis has also been suggested in pancreatic and ovarian cancers [7]. Interestingly, CLDN4 binds to integrins and promotes survival signals and stemness even when tight junctions are not formed [5]. In addition, reduced expression of CLDN4 in tumors is also associated with metastasis as they reflect epithelial-mesenchymal transition (EMT) [5,8]. Maintenance of the intratumor microenvironment by CLDN4 promotes malignant phenotypes in cancer through retention of growth factors and suppression of oxidative stress [3,9].

Epigenetic alterations play a major role in carcinogenesis and cancer progression in various malignancies [10,11,12]. DNA methylation, histone modifications, chromatin remodeling, and microRNA are considered useful indicators of cancer development and progression [13]. Epigenetic alteration in the regulation of CLDN4 expression has recently been reported. In gastric, bladder, and colorectal cancers, hypermethylation of the CpG sequence in the *CLDN4* gene promoter region leads to decreased CLDN4 expression [14,15,16]. In contrast, hypermethylation of the *CLDN4* gene and overexpression of CLDN4 have been reported in ovarian cancer [17]. In BUC, CLDN4 overexpression is associated with cancer progression [3]; however, its epigenetic status remains unclear. Therefore, in this study, we investigated the *CLDN4* promoter DNA methylation status and corresponding protein expression in BUC and examined its association with the malignant phenotype.

## 2. Results

### 2.1. DNA Methylation in the CLDN4 Promoter in BUCs

**Figure 1:**
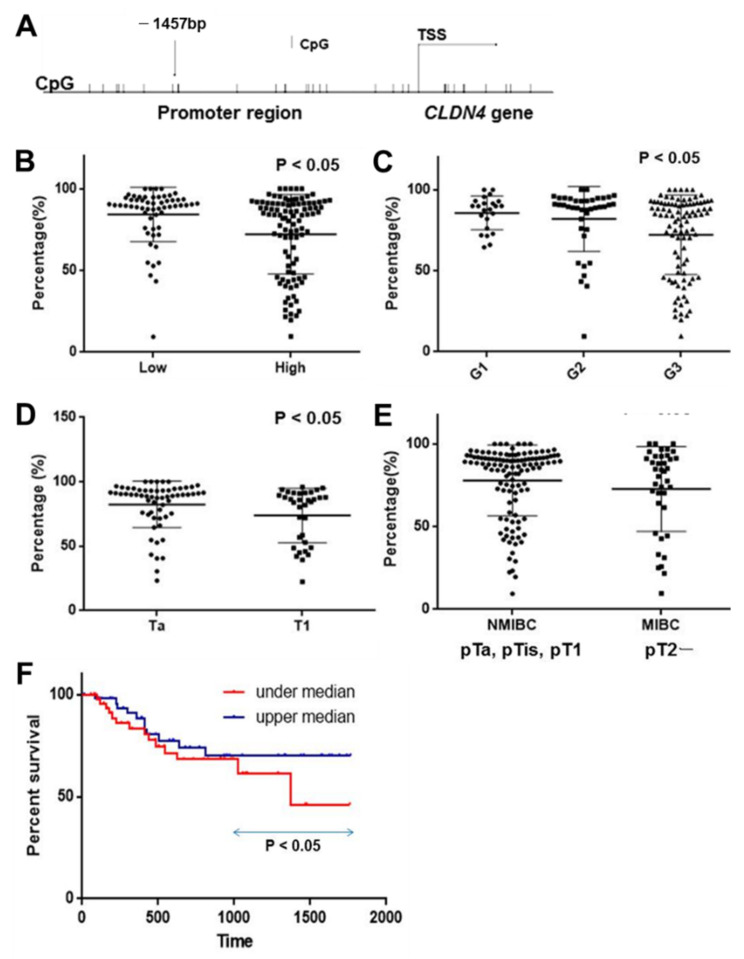
Hypomethylation of promoter DNA in *CLDN4* gene in 157 cases of BUCs. (**A**) Examined CpG island in *CLDN4* promoter region. (**B**) Comparison of *CLDN4* promoter methylation between low-grade and high-grade BUCs. (**C**) Comparison of *CLDN4* promoter methylation among G1, G2, and G3 BUCs. (**D**) Comparison of *CLDN4* promoter methylation between pTa and pT1 BUCs. (**E**) Comparison of *CLDN4* promoter methylation between NMIBCs and MIBCs. (**F**) Comparison of overall survival between *CLDN4* promoter methylation low (under median) and high (upper median) cases. Grade (high, low, or G1, G2, or G3) and primary tumor (pT; pTa, non-invasive papillary BUC; pT1, invasion into subepithelial layer) are classified according to the TNM classification system [18]. The data show the mean ± SD, wherein the SD was calculated by Student’s *t*-test. Survival analysis was performed by Kaplan–Meier method with statistical test by generalized Wilcoxon test. CLDN, claudin; BUC, bladder urothelial carcinoma; TSS, transcription starting site; percentage, frequency of CpG methylation; NMIBC, non-muscle invasive BUC; MIBC, muscle invasive BUC.

The promoter region of the *CLDN4* gene contains several CpG sites, as shown in Figure 1A. In this study, we examined the methylation status of the CpG site located at the 1457 base pair (bp) upstream of the transcription start site (TSS). Methylation of this *CLDN4* promoter CpG site was examined using tissues from 157 BUC cases (Figure 1B–E, Table 1). The methylation rate in non-neoplastic bladder urothelium was 92 ± 5%, whereas the BUC samples were significantly hypomethylated at 72 ± 19% (*p* < 0.0001). Furthermore, the methylation rate was lower in high-grade than in low-grade BUC (Figure 1B), and in the order of G3, G2, and G1 (Figure 1C, Table 1). With regard to wall invasion (pT factor) (Table 1), the methylation rate was lower in cases with subepithelial invasion (pT1) than in cases with non-invasive carcinoma (pTa), as well as lower in cases with muscle layer invasion (MIBC) than in cases with non-muscle layer invasion (NMIBC). When survival was examined in the top 50% (high methylation) and the bottom 50% (low methylation) groups, the overall survival was significantly poorer in the low methylation cases (Figure 1F). Thus, *CLDN4* hypomethylation correlated with BUC malignant phenotype.

### 2.2. CLDN4 Promoter DNA Methylation and Gene Expression

**Figure 2:**
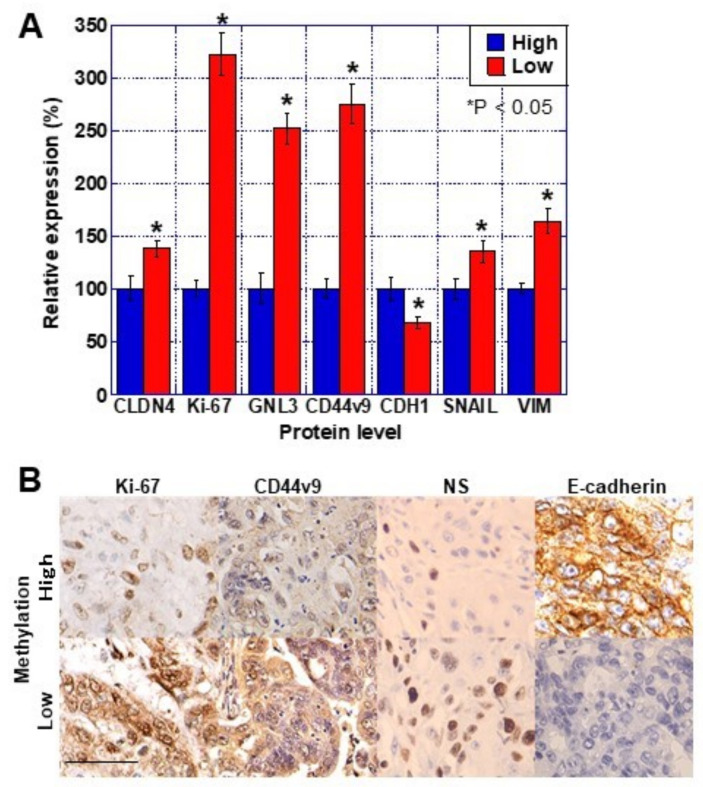
Levels of malignant phenotype-associated proteins in 23 cases of BUCs. Protein expression in the 23 BUC cases shown in Table 2 (11 high-methylation and 12 low-methylation cases) were examined by (**A**) ELISA and (**B**) immunohistochemistry. Relative protein levels of CLDN4, Ki-67 (proliferation), GNL3 and CD44v9 (stemness), and CDH1, SNAIL, and vimentin (EMT). Error bar indicates the SD calculated by Student’s *t*-test. Scale bar, 50 μm. CLDN, claudin; BUC, bladder urothelial carcinoma; VIM, vimentin; EMT, epithelial-mesenchymal transition.

Next, we examined *CLDN4* promoter methylation and expression of *CLDN4*, stem cell-related genes, and EMT-related genes in 23 invasive BUC samples from the above 157 cases from which mRNA extraction was possible. The comparison of the clinicopathological factors and *CLDN4* promoter methylation among the 23 cases is shown in Table 2. The hypomethylated cases showed an infiltrative invasive pattern of cancer, greater frequency of vascular invasion, and more local recurrences than the hypermethylated cases. In terms of gene expression, the hypomethylated cases showed higher *CLDN4* expression and proliferative activity (Ki-67) compared to the hypermethylated cases (Figure 2A). The stemness markers *GNL3* and *CD44v9* showed higher expression in the hypomethylated cases than in the hypermethylated cases. Among the EMT-related genes, *CDH1* expression was decreased while that of SNAIL and vimentin was increased in the hypomethylated cases compared to the hypermethylated cases. Immunostaining confirmed that these changes at the mRNA level were also reflected at the protein level (Figure 2B). Thus, *CLDN4* hypomethylation was associated with increased CLDN4 expression, stemness, and EMT induction in BUC.

### 2.3. Effects of CLDN4 Promoter DNA Demethylation

**Figure 3:**
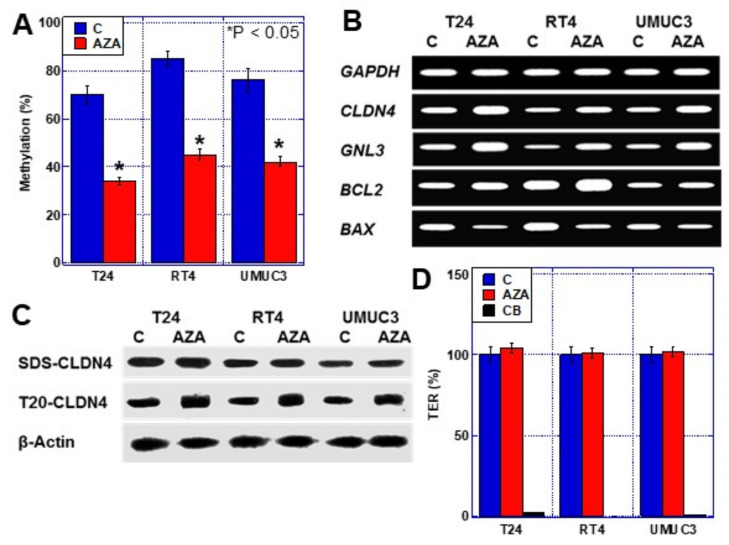
Effect of AZA treatment in three human BUC cells. (**A**) Frequency of methylation of *CLDN4* promoter in T24, RT4, and UMUC3 human BUC cells with or without AZA treatment. (**B**) Effect of AZA on mRNA expression. (**C**) Effect of AZA on CLDN4 protein expression and tight junction integrity in T24 cells. (**D**) Effect of AZA on TER in T24 cells. Error bar indicates SD calculated by Student’s *t*-test. CLDN, claudin; BUC, bladder urothelial carcinoma; AZA, 5-aza-2-deoxycytidine; SDS-CLDN4, 0.5% sodium dodecyl sulfate-solubilized CLDN4 (whole CLDN4); T20-CLDN4, 0.5% Tween 20-solubilized CLDN4 (tight junction unintegrated CLDN4); TER, transepithelial electrical resistance; CB, cytochalasin B.

The results indicated that the *CLDN4* promoter DNA is hypomethylated in BUC. Next, we treated three human BUC cell lines with the demethylating agent aza-2′-deoxycytidine (AZA) and examined its effects (Figure 3). In BUC cells, AZA treatment reduced *CLDN4* promoter DNA methylation rate to approximately 50%. Furthermore, *GNL3*, the stemness marker, and *BCL2*, the anti-apoptotic factor, were upregulated, while *BAX*, the pro-apoptotic factor, was downregulated upon AZA treatment (Figure 3B).

Examination of the protein level of whole CLDN4 (sodium dodecyl sulfate-solubilized CLDN4; SDS-CLDN4) following increased expression of *CLDN4* mRNA by AZA treatment showed no change (Figure 3C). In contrast, protein levels of tight junction-unintegrated CLDN4 (Tween 20-solubilized CLDN4; T20-CLDN4) were increased in the three types of BUC cells. Furthermore, when the function of the tight junction was examined by transepithelial electric resistance (TER) assay, the TER was not found to be altered by AZA treatment (Figure 3D). Thus, excess CLDN4 expression by *CLDN4* promoter DNA hypomethylation increased only the T20-CLDN4 levels and promoted stemness and anti-apoptotic survival.

### 2.4. Effects of CLDN4 Knockdown

**Figure 4:**
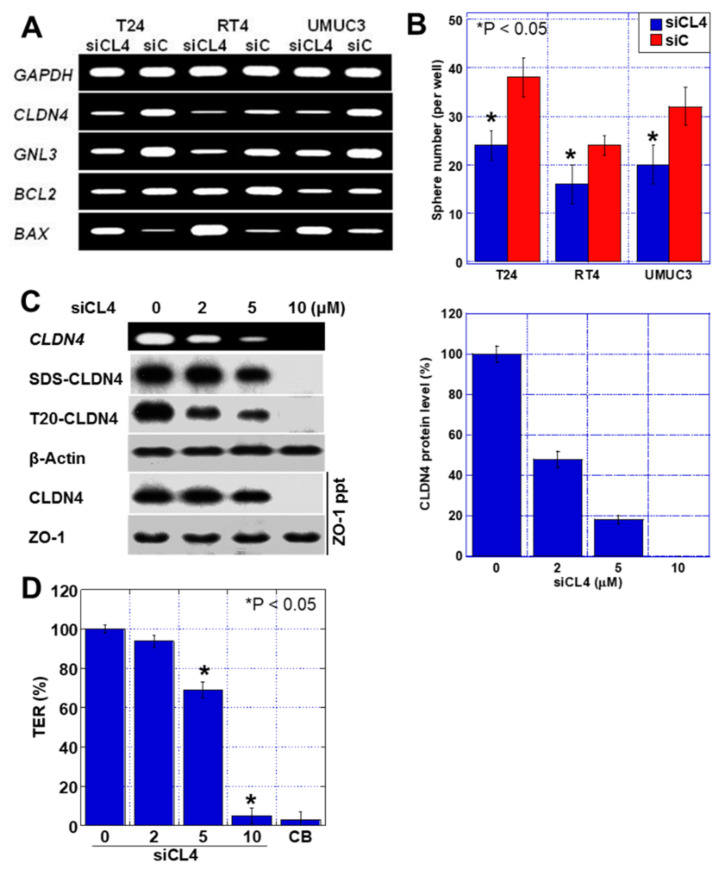
Effect of *CLDN4* knockdown in three human BUC cells. (**A**) Effect of *CLDN4* knockdown on mRNA expression. (**B**) Effect of *CLDN4* knockdown on sphere formation. (**C**) Effect of *CLDN4* knockdown on CLDN4 expression and tight junction integrity. (Right) Semi-quantification of CLDN4 protein levels in siCL4-treated T24 cells. (**D**) Effect of *CLDN4* knockdown on TER in T24 cells. Error bar indicates SD calculated by Student’s *t*-test. CLDN, claudin; BUC, bladder urothelial carcinoma; si, short interference RNA; siC, control siRNA; siCL4, siRNA for CLDN4; SDS-CLDN4, 0.5% sodium dodecyl sulfate-solubilized CLDN4 (whole CLDN4); T20-CLDN4, 0.5% Tween 20-solubilized CLDN4 (tight junction unintegrated CLDN4); ppt, immunoprecipitant; TER, transepithelial electrical resistance; CB, cytochalasin B.

Next, *CLDN4* was knocked down by short interference RNA (siRNA), and its effects were examined (Figure 4). *CLDN4* knockdown resulted in a decreased expression of *CLDN4*, stemness marker *GNL3*, and anti-apoptotic factor *BCL2*, and increased expression of the pro-apoptotic factor *BAX* (Figure 4A). In addition, *CLDN4* knockdown decreased the sphere-forming ability of the cells, indicating decreased stemness (Figure 4B). Further, we changed the CLDN4 expression levels in T24 cells using various siRNA concentrations, followed by measurement and comparison of the levels of SDS-CLDN4 and T20-CLDN4 and TER (Figure 4C,D). The 2 µM siRNA treatment reduced T20-CLDN4, whereas SDS-CLDN4 levels were largely unchanged and ZO-1-bound CLDN4 levels were preserved. In this knockdown condition, no significant decrease was observed in TER. In contrast, 5 μM siRNA treatment decreased SDS-CLDN4 and ZO-1-bound CLDN4 levels, along with a decrease in TER. Thus, T20-CLDN4 was not linked to tight junction function, implying that it might be the excess relative to the amount of CLDN4 that is required to maintain tight junction function.

### 2.5. Non-Tight Junction CLDN4 Binding to Integrinβ1

**Figure 5:**
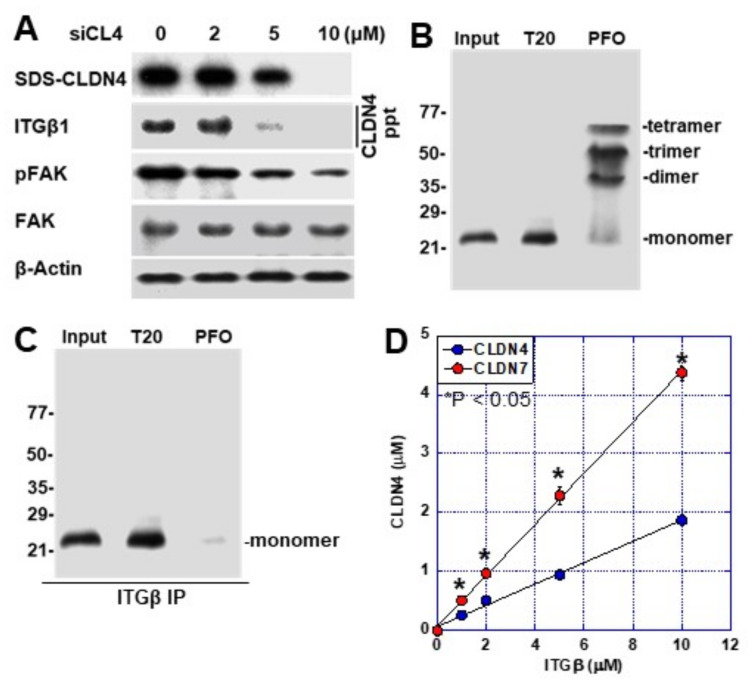
Physical association between CLDN4 and integrin β1 in T24 cells. (**A**) Effect of *CLDN4* knockdown on CLDN4–ITGβ1 binding and FAK phosphorylation. (**B**) CLDN4 detected by immunoblotting using 0.5% Tween 20-solubilized lysate (T20) or 0.5% perfluoro-octanoic acid-soluble lysate (PFO). (**C**) CLDN4 bound to ITGβ1 detected by immunoprecipitation. (**D**) Comparison of the affinity to ITGβ1 between CLDN4 and CLDN7. Error bar indicates SD calculated by Student’s *t*-test from three independent trials. CLDN, claudin; BUC, bladder urothelial carcinoma; si, short interference RNA; siC, control siRNA; siCL4, siRNA for CLDN4; ITGβ1, integrin β1; FAK, focal adhesion kinase; pFAK, phosphorylated FAK. SDS-CLDN4, 0.5% sodium dodecyl sulfate-solubilized CLDN4 (whole CLDN4); T20, 0.5% Tween 20-solubilized CLDN4 (tight junction unintegrated CLDN4); PFO, 0.5% perfluoro-octanoic-solubilized CLDN4 (tight junction integrated CLDN4); ppt, immunoprecipitant.

We previously reported that CLDN4 acts as a ligand for integrin β1 and is involved in the subsequent signal formation [5]. Here, we examined the association between CLDN4 and integrin β1 in T24 cells (Figure 5). The levels of CLDN4-bound integrin β1 and phosphorylated FAK both decreased upon knockdown of *CLDN4*. Next, we examined binding of integrin β1 to T20-CLDN4 and tight junction-integrated CLDN4 (perfluoro-octanoic acid-soluble CLDN4; PFO-CLDN4) and confirmed that it binds only to T20-CLDN4 (Figure 5B,C). In the case of PFO-CLDN4, dimer, trimer, and tetramer were extracted along with the monomer (Figure 5B). In contrast, extraction of T20-CLDN4 showed only the monomer. When this extract was mixed with recombinant integrin β1 and incubated for binding, only the CLDN4 monomer bound to integrin β1 (Figure 5C). Since the binding of CLDN7 to integrin β1 has been reported [19], the binding of integrin β1 with CLDN4 and CLDN7 was compared (Figure 5D). The affinity between integrin β1 and CLDN4 was found to be approximately 40% of that observed with CLDN7.

### 2.6. Effects of Integrin Activation by CLDN4

**Figure 6:**
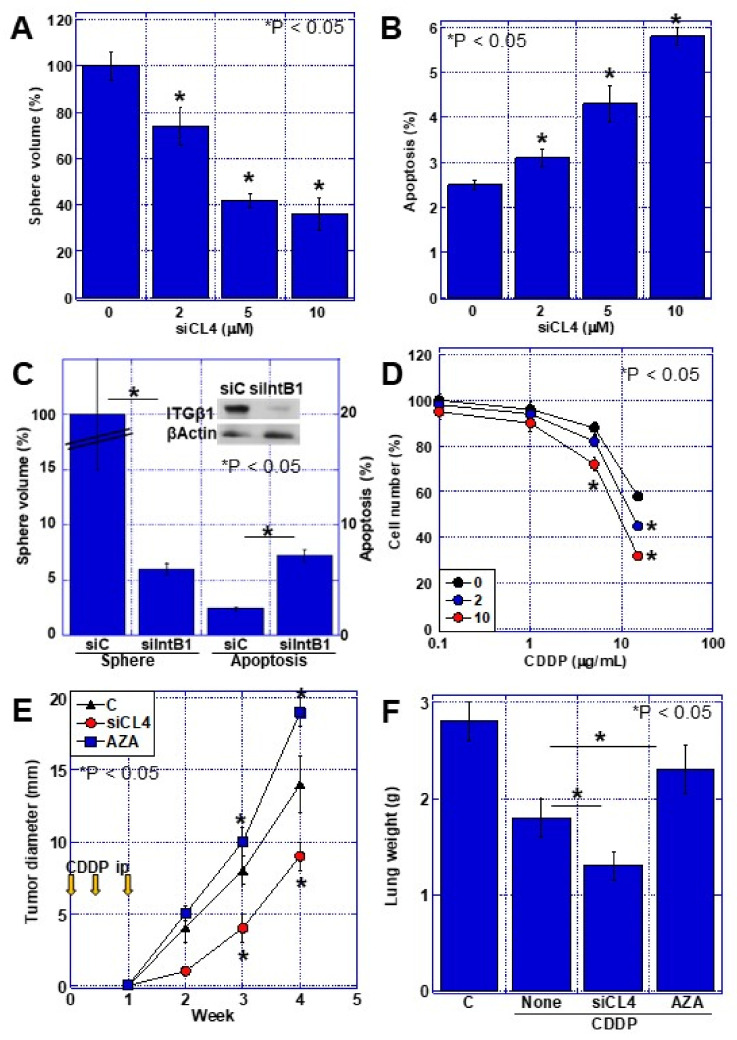
Effect of CLDN4-integrin β1 interaction on malignant phenotypes in T24 BUC cells. (**A**) Effect of *CLDN4* knockdown on sphere formation. (**B**) Effect of *CLDN4* knockdown on apoptosis. (**C**) Effect of *ITGB1* knockdown on sphere formation and apoptosis. (**D**) Effect of *CLDN4* knockdown on chemosensitivity to CDDP. (**E**) Effect of CDDP on tumor growth of T24 cells pretreated with control vehicle (**C**), CLDN4 siRNA (siCL4), or 5-AZA (AZA). (**F**) Effect of CDDP on lung metastasis of T24 cells pretreated with control vehicle (**C**), CLDN4 siRNA (siCL4), or 5-AZA (AZA). Error bar indicates SD calculated by Student’s *t*-test from three independent trials or 5 mice. CLDN, claudin; BUC, bladder urothelial carcinoma; si, short interference RNA; siC, control siRNA; siCL4, siRNA for CLDN4; siIntB1, siRNA for integrin β1; ITGβ1, integrin β1; CDDP, cisplatin.

Finally, the effect of integrin β1 activation by CLDN4 was examined in T24 cells (Figure 6). We examined the effects on sphere formation and apoptosis in a *CLDN4* knockdown system (Figure 6A,B). Upon decreasing *CLDN4* expression by knockdown, the sphere volume decreased while apoptosis increased. Knocking down integrin β1 resulted in greater changes in both sphere formation and apoptosis than *CLDN4* knockdown (Figure 6C). Furthermore, knockdown of *CLDN4* increased the sensitivity of the cells to the drug cisplatin (CDDP) (Figure 6D). In a subcutaneous mouse tumor model, CDDP suppressed tumor growth after *CLDN4* knockdown, whereas increasing CLDN4 expression by AZA treatment enhanced tumor growth. In a mouse model of lung metastasis induced by tail vein inoculation, *CLDN4* knockdown suppressed lung metastasis, whereas increasing CLDN4 expression by AZA treatment enhanced lung metastasis.

## 3. Discussion

Our analysis of human BUC cases revealed that *CLDN4* promoter DNA hypomethylation causes CLDN4 overexpression in BUC. We also found that CLDN4 overexpression increased non-tight junction CLDN4 expression, resulting in increased stemness, likely via activation of integrin β1, suppression of apoptosis, decrease in drug sensitivity, and promotion of tumor growth and metastasis.

The involvement of epigenetics in CLDN expression has been reported for CLDN1 [16,20,21], CLDN2 [22], CLDN3 [23,24,25], CLDN6 and 9 [26], CLDN7 [27], and CLDN11 [28]. Epigenetic involvement has also been reported for CLDN4 [14,24], wherein, similar to this study, CLDN4 overexpression by promoter hypomethylation was reported in gastric cancer [14].

This study shows that decreased *CLDN4* promoter DNA methylation results in increased CLDN4 expression. In our previous study, CLDN4 overexpression correlated with cancer progression in BUC [3]. In this study, we also found a correlation between *CLDN4* promoter DNA hypomethylation and CLDN4 overexpression and BUC grade or invasion. In cases with *CLDN4* promoter DNA hypomethylation, accelerated proliferation, increased stemness, and enhanced EMT phenotype were observed. Furthermore, *CLDN4* promoter DNA hypomethylation of BUC cell lines by 5-AZA treatment resulted in enhanced CLDN4 expression, increased stemness, and a higher expression of anti-apoptotic factors. In contrast, knockdown of *CLDN4* showed the opposite results. In our previous report, CLDN4 overexpression increased malignancy by promoting resistance to anticancer drugs [3]; however, our results show that CLDN4 overexpression increases cancer malignant phenotypes in situations without usage of anticancer drugs.

Until now, CLDN4 expression has been implicated as an epithelial marker, and its reduction suggests the EMT phenotype [29]. In this study, we examined the association between CLDN4 overexpression and function of tight junctions. Interestingly, the excess expression of CLDN4 above the expression level sufficient to retain tight junction function for TER generated a monomer of CLDN4 that is not integrated into tight junctions. Furthermore, the non-tight junction CLDN4 promoted stemness, EMT phenotype, proliferation, and anti-apoptotic survival. We have previously reported roles of non-tight junction CLDN4: FAK activation by CLDN4 on the plasma membrane [5] and YAP activation by migration into the cytoplasm and nucleus [30,31]. The diversity of non-tight junction functions of CLDIN-4 has attracted much attention [32], and our studies have shown that CLDN4 overexpression is associated with malignant phenotypes in cancer cells through a variety of mechanisms.

Our results revealed that the increased BUC malignant phenotypes due to CLDN4 overexpression is due to increased levels of non-tight junction CDLN4, which promotes stemness through the activation of integrin β1. CLDN7 has been previously reported to bind integrin β1 and result in downstream FAK phosphorylation [19,33]. Moreover, we have previously shown that CLDN4, as well as CLDN7, binds to integrin β1 and activates FAK in BUC in gastric cancer [5]. In this study, CLDN4 showed approximately 40% of the affinity of CLDN7 for integrin β1. Integrin β1 is known to promote stemness through downstream FAK activation [34,35]. Alterations in the association of cancer cells with the stroma [36] and Notch signaling have been reported as the underlying mechanisms [35]. In the present study, binding CLDN4 to integrin β1 resulted in FAK phosphorylation and increased sphere-forming capacity. As a result of this, we observed increased stemness, decreased apoptosis, increased drug resistance, and increased cancer metastatic potential.

In the present study, CLDN4 promoter hypomethylation induced CLDN4 overexpression in bladder cancer. However, the cause of the hypomethylation could not be clarified. BUC is associated with abnormal DNA methylation, and disruption of the methylation pattern establishment mechanism may result in deregulation of DNA methyltransferases and demethylases and altered metabolism of methyl groups [37]. In BUCs, several methyltransferases are upregulated [38,39,40]; their enhanced activity may result in depletion of methyl donors, thus potentially inducing an hypomethylation of gene promoters.

Thus, the results suggest that *CLDN4* promoter DNA hypomethylation is the cause of CLDN4 overexpression in BUC, and that overexpression results in increased non-tight junction CLDN4, which promotes cancer stemness through integrin β1 activation and consequent increased malignant potential. Furthermore, an increased CLDN4 level correlates with tumor virulence through various mechanisms, including increased drug resistance by tight junction barrier function, increased EMT phenotype, and increased stemness. These findings suggest that *CLDN4* promoter DNA methylation may be a new marker of bladder cancer malignancy and may be applied to develop novel therapeutic targets.

## 4. Materials and Methods

### 4.1. Surgical Specimens

In total, 157 paired tissue samples of tumor and the corresponding normal-appearing tissue adjacent to the tumor were obtained from patients with BUC during the surgical procedure (transurethral resection or radical cystectomy). Corresponding normal-appearing tissues were judged macroscopically or endoscopically and dissected. Half of the tissues were used for pathological examination; if the tissue included cancer tissue, the section was excluded from the analyses. Control tissue samples of normal urothelia were obtained from patients without BUC. Tumors were staged according to the UICC TNM classification system [18]. All collected tissues were frozen and stored at −80 °C until use for DNA extraction.

As written informed consents were not obtained from the patients, any identifying information was removed from the samples prior to analysis in order to ensure strict privacy protection (unlinkable anonymization). All procedures were performed in accordance with the Ethical Guidelines for Human Genome/Gene Research issued by the Japanese Government and were approved by the Ethics Committee of Nara Medical University (approval number 937, 20 October 2010).

### 4.2. Cells and Reagents

T24, RT4, and UMUC3 human BUC cell lines were purchased from the American Type Culture Collection (ATCC; Manassas, VA, USA). Cells were cultured in Dulbecco’s modified Eagle’s medium supplemented with 10% fetal bovine serum at 37 °C in 5% CO_2_. Cell growth was assessed using tetrazolium (MTT) dye assay as previously described [41]. CDDP was purchased from Novaplus (Bedford, OH, USA).

### 4.3. DNA Extraction

DNA was extracted using conventional extraction methods [42]. DNA (2 μg) was treated with sodium bisulfite using an Epitect Bisulfite Kit (Qiagen) according to the manufacturer’s protocol and resuspended in 40 μL of distilled water for subsequent use.

### 4.4. The Primer Set for Pyrosequencing

The genomic sequences of CLDN4 gene (NCBI Reference Sequence: NG_012868.1) obtained from the National Center for Biotechnology Information (NCBI). DataBase of CpG islands and Analytical Tool (DBCAT) was used to identify CpG sites. The primer set for pyrosequence was designed by PyroMark Assay Design Software 2.0 (Qiagen, Hilden, Germany). PCR primers were designed to include the -1457 CpG site, and their sequences are: forward: 5′-GAA TTG GAT ATA TAG TTA TTA GTG TTG-3′ and reverse: 5′-CCA ACC ATA CTA AAA ACT CTA CA-3′. The sequencing primer was 5′-AGT TAT TAG TGT TGG ATA ATG-3′ (synthesized by Sigma Genosys, Ishikari, Japan).

### 4.5. Bisulfite Pyrosequencing

DNA methylation status of *CLDN4* CpG sites was assessed by pyrosequencing using Pyrosequencing 96HS (Biotage, Uppsala, Sweden) and PyroMark Q24 (Qiagen) according to the manufacturer’s protocol. Reaction volumes of 30 μL contained 5× GoTaq buffer, 1.5 U GoTaq Hot Start Polymerase (Promega Biosciences, San Louis Obispo, CA, USA), 1 μM of primers, and 500 nM of dNTPs. PCR conditions were as follows: 95 °C for 3 min; 35 cycles of 95 °C for 30 s, 65 °C for 30 s, and 72 °C for 30 s, and a final extension step at 72 °C for 4 min. The pyrosequenced product including the CpG region was assayed on the Illumina GoldenGate Panel.

### 4.6. Small Interfering RNA

Stealth Select RNAi (siRNA) targeting mouse *Hmgb1* and rat *Hmgb1* were purchased from Sigma (St. Louis, MO, USA). AllStars Negative Control siRNA was used as a control (Qiagen). The cells were transfected with 10 µM of siRNA using Lipofectamine 3000 (Thermo Fisher, Tokyo, Japan) according to the manufacturer’s recommendations.

### 4.7. Enzyme-Linked Immunosorbent Assay (ELISA)

ELISA kits were used for measuring protein concentrations of CLDN4, Ki-67, GNL3, CD44v9, SNAIL, and vimentin (Table 3), according to the manufacturer’s instructions.

### 4.8. Immunohistochemistry

Consecutive sections of 4 μm of BUC tissues were immunohistochemically stained using 0.2 µg/mL of primary antibodies using a previously described immunoperoxidase technique [39]. Secondary antibodies (peroxidase-conjugated antibodies, MBL, Nagoya, Japan) were used at a concentration of 0.2 µg/mL. Tissue sections were color-developed with diamine benzidine hydrochloride (DAKO, Glastrup, Denmark) and counterstained with Meyer’s hematoxylin (Sigma). The primary antibodies used are listed in Table 3.

### 4.9. Protein Extraction

For preparing whole cell lysate, BUC cells were washed twice with cold phosphate-buffered saline, harvested, and lysed with 0.1% sodium dodecyl sulfate (SDS), 0.5% Tween 20-, or 0.5% perfluoro-octanoic acid (PFO)-added RIPA-buffer (Thermo Fisher Scientific, Tokyo, Japan) supplemented with protease inhibitor cocktail (Promega) [43]. SDS solubilizes whole CLDN4 protein, Tween-20 solubilizes the CLDN4 monomer, i.e., non-tight junction CLDN4, and PFO solubilizes CLDN4 polymer, i.e., tight junction CLDN4 [44]. Protein assay was performed using a Protein Assay Rapid Kit (Wako Pure Chemical, Osaka, Japan).

### 4.10. Immunoblot Analysis

Whole-cell lysates of BUC cells were prepared as previously described [43]. Lysates (20 μg) were subjected to immunoblot analysis using SDS polyacrylamide gel electrophoresis (12.5%), followed by electrotransfer onto nitrocellulose filters. The filters were incubated with primary antibodies (diluted to 1/500 by 1x tris buffered saline with Tween 20 [TBS-T20, Thermo Fisher Pierce, Tokyo, Japan]) for 120 min at room temperature, and then washed with TBS-T20 3 timed at room temperature. The filters were incubated with primary antibodies (diluted to 1/500 by 1x tris buffered saline with tween 20 [TBS-T20, Thermo Fisher Pierce, Tokyo, Japan]) for 120 min at room temperature, and then washed with TBS-T20 3 timed at room temperature. Filtered were incubated with peroxidase-conjugated IgG antibodies (MBL, diluted to 1/200 by TBS-T20) for 120 min at room temperature, and then washed with TBS-T20 3 timed at room temperature. Anti-tubulin antibody was used to assess the levels of protein loaded per lane (Oncogene Research, Cambridge, MA, USA). The immune complex was visualized using an Enhanced Chemiluminescence Western-blot detection system (Amersham, Aylesbury, UK). The primary antibodies used are listed in Table 3. Images were captured on a computer and the signal strength was measured using NIH ImageJ software (version 1.52, Bethesda, MD, USA).

### 4.11. Immunoprecipitation

Immunoprecipitation was performed according to a method described previously [45]. Lysates were pre-cleaned in lysis buffer with protein A/G agarose (Santa Cruz) for 1 h at 4 °C and subsequently centrifuged. The supernatants were incubated with a precipitation antibody [either against ZO-1 or CLDN4 (4D3)] and protein A/G agarose for 3 h at 4 °C. Precipitates were collected via centrifugation, washed five times with lysis buffer, solubilized with sample buffer (Sigma, 40 µg), and subjected to an immunoblot analysis with antibodies against CLDN4 (4D3), integrin β1, and/or ZO-1. The antibodies used are listed in Table 3.

### 4.12. Affinity Assay

Recombinant human integrin β1 (Abcam, Cambridge, UK, the used amount is shown in Figure 5D) was mixed with recombinant human CLDN4 (Abcam) or recombinant human CLDN7 (Cloud Cone, Tokyo, Japan) in equal amounts in a binding buffer containing 20 mM of Tris-HC1, 150 mM of NaC1, l mM of CaCI, and 35 mg/mL of bovine serum albumin and incubated for 2 h at 30 °C. The reaction solution was subjected to immunoprecipitation with anti-integrin β1 antibody. CLDN4 or CLDN7 in the immunoprecipitant was measured by ELISA kit. The antibodies and ELISA kits used are listed in Table 3.

### 4.13. Reverse Transcription-Polymerase Chain Reaction (RT-PCR)

To assess human *CLDN4* mRNA expression, RT-PCR was performed with 0.5 µg of total RNA extracted from BUC cells using an RNeasy kit (Qiagen). The primer sets used are shown in Table 4. Primers were synthesized by Sigma Genosys. The *GAPDH* mRNA was also amplified for use as an internal control.

### 4.14. Sphere Assay

Cells (1000 cells per well) were seeded onto uncoated bacteriological 35-mm dish (Coning, Coning, NY, USA) with 3D Tumorsphere Medium XF (Sigma). After 7 days, sphere images were captured on a computer, and the sphere volume was measured using NIH ImageJ software (version 1.52, Bethesda, MD, USA).

### 4.15. Apoptosis Assay

Apoptosis was assessed via the examination of 1000 cells, which were stained with Hoechst 33342 dye (Life Technologies, Carlsbad, CA, USA), and viewed using a fluorescent microscope.

### 4.16. Transepithelial Electroresistance (TER) Assay

The cellZscope tight junction monitoring system (Fujifilm, Tokyo, Japan) was used to measure the TER of the cells. Briefly, 1 × 10^5^ cells were seeded onto the provided insert and allowed to form multiple cell layers. Thereafter, the TER was measured using the system as per the manufacturer’s instructions. A negative control was generated, in which tight junction formation was impaired via treatment with cytochalasin B (CB; 10 μM, Wako).

### 4.17. Animals

Four-week-old male BALB/c Slc-nu/nu mice were procured from SLC Japan (Shizuoka, Japan). The mice were maintained according to the institutional guidelines approved by the Committee for Animal Experimentation of Nara Medical University, in accordance with the current regulations and standards of the Ministry of Health, Labor, and Welfare (Approval no. 11356, 1 February 2016).

### 4.18. Animal Tumor Models

To establish a subcutaneous tumor model, T24 cancer cells (1 × 10^7^) were inoculated into the scapular subcutaneous tissues of nude mice (5 mice in each group). For the lung metastasis model, T24 cancer cells (1 × 10^6^) were inoculated into the caudal vein (5 mice in each group). The lungs of the mice were observed for 4 weeks following inoculation. The lungs were sliced into 2 mm thick sections and the metastatic foci were counted under a stereomicroscope (Nikon, Tokyo, Japan). CDDP (3 mg/kg) was administered into the peritoneal cavity on days 1, 3, and 7.

### 4.19. Statistical Analysis

Statistical significance was calculated using the chi-squared test, Fisher’s square test, and Kruskal–Wallis test with InStat software (GraphPad, Los Angeles, CA, USA). Statistical significance was defined as a two-sided *p*-value of <0.05.

## Figures and Tables

**Table 1:** Methylation levels of promoter CpG of *CLDN4* gene in 157 BUC samples.

Parameter		*n*	Methylation (%)	*p*
Normal urothelium		20	92 ± 5	
BUC		157	72 ± 19	<0.0001
Grade ^(1)^	G1	23	81 ± 12	
	G2	39	77 ± 24	
	G3	95	68 ± 20	0.0057
pT ^(2)^	pTa	61	82 ± 19	
	pTis	23	75 ± 20	
	pT1	34	73 ± 23	
	pT2	39	69 ± 23	0.0215
Muscle	NMIBC	118	78 ± 21	
invasion	MIBC	39	69 ± 23	0.0249

BUC, bladder urothelial carcinoma; CLDN, claudin; NMIBC, non-muscle invasion bladder cancer; MIBC, muscle invasion bladder cancer. ^(1)^ Histological grade; G1, well differentiated; G2, moderately differentiated; G3, poorly differentiated according to TNM classification system [18]. ^(2)^ pT, primary tumor; pTa, non-invasive papillary urothelial carcinoma; pTis, carcinoma in situ; pT1, subepithelial invasion; pT2, muscle invasion according to TNM classification system [18].

**Table 2:** Comparison of pathological parameters between CLDN4-methylation high and low cases.

Parameter		CLDN4 Methylation ^(1)^		*p*
		High	Low	
Number		11	12	
Grade ^(2)^	Low	1	0	NS
	High	10	12	
pT ^(3)^	pT1	10	10	NS
	pT2	1	2	
INF ^(4)^	a/b	11	5	0.0046
	c	0	7	
Ly/V ^(5)^	-	11	4	0.0006
	+	0	9	
Reurrence ^(6)^		3	10	0.0123

^(1)^ Methylation high, cases belonging to the upper 50% of methylation percentage; methylation low, cases belonging to the lower 50% of methylation percentage. ^(2)^ Histological grade low, G1-2; high, G3 according to TNM classification system [18]. ^(3)^ pT, primary tumor; pT1, subepithelial invasion; pT2, muscle invasion according to TNM classification system [18]. ^(4)^ INF, tumor infiltrative pattern into the surrounding tissues; INFa, expanding growth with a distinct border; INFb, intermediate pattern between INFa and INFc; INFc, infiltrative growth with no distinct border. ^(5)^ Ly/V, capillary invasion; Ly, lymphatic invasion; V, venous invasion. ^(6)^ Recurrence, local recurrence after transurethral resection.

**Table 3:** Antibodies and ELISA kits used.

Antibody	Species	Provider	City and Country
CLDN4	human	clone 4D3	[3]
ZO-1	human	Abcam	Cambridge, MA, USA
ITGβ1	human	Santa-Cruz Biotechnology	Santa Cruz, CA, USA
pFAK (pTyr397)	human	Boster Immunoleader	Pleasanton, CA, USA
FAK	human	Santa-Cruz Biotechnology	Santa Cruz, CA, USA
Ki-67	human	DAKO-Agilent	Santa Clara, CA, USA
CD44v9	human	Cosmobio	Carlsbad, CA, USA
Nucleostemin	human	Santa-Cruz Biotechnology	Santa Cruz, CA, USA
E-cadherin	human	DAKO-Agilent	Santa Clara, CA, USA
βActin	human	Zymed Laboratories Inc.	South San Francisco, CA, USA
**ELISA**	**Species**	**Provider**	**City and country**
CLDN4	human	MyBiosource, Inc.	San Diego, CA, USA
CLDN7	Human	Wuhan Huamei Biotech	Wuhan, China
Ki-67	human	Abcam	Cambridge, MA, USA
GNL3	human	antibodies-online GmbH	Aachen, Germany
CD44v9	human	Elabscience	Houston, TX, USA
CDH1	human	Abcam	Cambridge, MA, USA
SNAIL	human	Biocompare	South San Francisco, CA, USA
Vimentin	human	Abcam	Cambridge, MA, USA

**Table 4:** Primer sets used.

Gene Symbol	Species	Accession No.	Site	Sequence
*CLDN4*	human	NM_001305.4	Upper	CTC CAT GGG GCT ACA GGT AA
			Lower	AGC AGC GAG TCG TAC ACC TT
*GNL3*	human	BC001024.2	Upper	ATT GCC AAC AGT GGT GTT CA
			Lower	AAT GGC TTT GCT GCA AGT TT
*BCL2*	human	M13994.1	Upper	ACG ACA ACC GGG AGA TAG TG
			Lower	CAT CCC AGC CTC CGT TAT CC
*BAX*	human	L22473.1	Upper	CAT GAA GAC AGG GGC CCT TT
			Lower	CTT CCA GAT GGT GAG CGA GG
*GAPDH*	human	BC025925.1	Upper	GAG TCA ACG GAT TTG GTC GT
			Lower	TTG ATT TTG GAG GGA TCT CG

## Data Availability

Not applicable.

## Abbreviations

TJ	tight junction
CLDN4	claudin-4
BUC	bladder urothelial carcinoma
EMT	epithelial-mesenchymal transition
AZA	aza-2′-deoxycytidine
SDS	sodium dodecyl sulfate
T20	tween 20
TER	transepithelial electric resistance
PFO	perfluoro-octanoic acid
CDDP	cisplatin
